# Deletion of the *Men1* Gene Prevents Streptozotocin-Induced Hyperglycemia in Mice

**DOI:** 10.1155/2010/876701

**Published:** 2011-01-17

**Authors:** Yuqing Yang, Haoren Wang, Xianxin Hua

**Affiliations:** ^1^Abramson Family Cancer Research Institute, Department of Cancer Biology, Abramson Cancer Center, University of Pennsylvania, Philadelphia, PA 19104, USA; ^2^Institute for Diabetes, Obesity, and Metabolism, University of Pennsylvania, Philadelphia, PA 19104, USA

## Abstract

Diabetes ultimately results from an inadequate number of functional beta cells in the islets of Langerhans. Enhancing proliferation of functional endogenous beta cells to treat diabetes remains underexplored. Here, we report that excision of the *Men1* gene, whose loss-of-function mutation leads to inherited multiple endocrine neoplasia type 1 (MEN1), rendered resistant to streptozotocin-induced hyperglycemia in a tamoxifen-inducible and temporally controlled *Men1* excision mouse model as well as in a tissue-specific *Men1* excision mouse model. *Men1* excision prevented mice from streptozotocin-induced hyperglycemia mainly through increasing the number of functional beta cells. BrdU incorporation by beta cells, islet size, and circulating insulin levels were significantly increased in *Men1*-excised mice. Membrane localization of glucose transporter 2 was largely preserved in *Men1*-excised beta cells, but not in *Men1*-expressing beta cells. Our findings suggest that repression of menin, a protein encoded by the *Men1* gene, might be a valuable means to maintain or increase the number of functional endogenous beta cells to prevent or ameliorate diabetes.

## 1. Introduction

Pancreatic beta cells are important for glucose sensing, insulin production and secretion, and therefore, are crucial for maintenance of blood glucose levels within the normal range [1, 2]. Both type 1 diabetes (T1D) and type 2 diabetes (T2D) ultimately result from an inadequate number of functional insulin-producing beta cells in the islets of Langerhans. T1D arises from dramatic autoimmune-induced beta-cell damage [1], while T2D develops due to insulin resistance as well as a relatively insufficient number of beta cells [3]. Insulin production is eventually insufficient for maintaining normal blood glucose levels in T1D or T2D, leading to hyperglycemia and secondary complications, including cardiovascular disease, kidney dysfunction, and blindness [1, 3, 4]. While therapies through immunomodulation or improving insulin sensitivity have shown promising effects on preventing or reversing hyperglycemia [5–8], how to promote proliferation or regeneration of functional endogenous beta cells to boost the number of functional beta cells needs to be further investigated. Only a handful of genes have been shown to affect development of hyperglycemia in diabetes mouse models. For example, overexpression of *Igf-1* in pancreatic beta cells prevents streptozotocin- (STZ-) induced diabetes [9]. Ablation of *p*27^*cip*1/*kip*1^, a cyclin-dependent kinase inhibitor, increases the number of beta cells and prevents development of hyperglycemia in *db/db* mice [10]. Deletion of *Lkb1*, a tumor suppressor activating AMP-activated protein kinase, promotes beta-cell proliferation and prevents glucose intolerance in high-fat diet-fed mice [11]. However, these genes have been shown to regulate proliferation of multiple types of cells [12, 13]. It is desirable to assess genes that preferentially affect beta-cell proliferation/regeneration in order to prevent or treat diabetes. 

Menin is a nuclear protein encoded by the *Men1* gene, which is mutated in familial multiple endocrine neoplasia type 1 (MEN1) patients [14]. Menin has been shown to preferentially repress cell proliferation in endocrine tissues including pancreatic beta cells [15, 16]. Although beta-cell proliferation and blood insulin levels are increased a long time after *Men1* is excised in beta cells [15, 17, 18], little is known as to whether *Men1* excision within a short time can prevent development of hyperglycemia in diabetes mouse models and its underlying mechanisms. 

 STZ is a glucose analog, and it selectively binds to glucose transporter 2 (GLUT2), the protein localized in the beta cell membrane, and is transported into beta cells [19, 20]. STZ induces beta cell damage, which mimics the loss of functional beta cells observed in T1D and T2D [20–23]. In the present study, we used a multiple low-dose streptozotocin- (MLD-STZ-) induced diabetes mouse model and determined the impact of *Men1* excision on STZ-induced hyperglycemia [19, 22]. We found that *Men1* excision prevented STZ-induced hyperglycemia, at least partly through promoting beta-cell proliferation, and preserving the number of functional beta cells and circulating insulin levels. These findings suggest that menin is crucial for regulating beta-cell proliferation *in vivo* and may perhaps be targeted for the prevention or treatment of diabetes.

## 2. Materials and Methods

### 2.1. Mice

*Me* *n*1^*l*/*l*^ *; Cre-ER* mice were generated by crossing mice with the floxed *Men1* (*Men*1^*l*/*l*^, FVB/129Sv) to mice harboring the Ubc9 promoter-driven *Cre-ERT2* (129Sv/C57BL6J, a gift from Dr. Eric Brown) [17, 24]. *Men*1^*l*/*l*^ mice, with exons 3 to 8 of *Men1* flanked by two loxP sites, were kindly provided by Dr. Francis Collins. *Men*1^*l*/*l*^ *; RIP-Cre* mice were generated by crossing floxed *Men1* mice with mice expressing *Cre *driven by the rat insulin-2 promoter (*RIP-Cre*, TgN(ins2-*cre*)25Mgn, Jackson Laboratory) [15]. Only male mice were used for the following experiments. The genotyping of mice was performed by PCR using mouse tail DNA as templates. For *Men1*: P1, 5′-CCCACATCCAGTCCCTCTTCAGCT-3′; P2, 5′-AAGGTACAGCAGAGGTCACAGAG-3′; P3, 5′-GACAGGATTGGGAATTCTCTTTT-3′. For *Cre-ERT2*: forward (F), 5′-ACACCAAAATTTGCCTGCATTACCGG-3′; reverse (R), 5′-TTTCCATGAGTGAACGAACCTGGT-3′. For* Rip-Cre*: F, 5′-GCGGTCTGGCAGTAA AAACTATC-3′; R, 5′-GTGAAACAGCATTGCTGTCACTT-3′. All mouse experiments were approved by the University Laboratory Animal Resource and the University of Pennsylvania Committee on Animal Care. The animal care was performed in accordance with institutional guidelines.

### 2.2. Excision of the Floxed *Men1* Locus Using Tamoxifen (TAM)

*Me* *n*1^*l*/*l*^; *Cre-ER* and their littermate controls were fed TAM (MP Biomedicals) at 200 mg/kg of body weight per day for two consecutive days, followed by one day off and then for another two consecutive days [18].

### 2.3. STZ-Induced Hyperglycemia

Hyperglycemia was induced by daily intraperitoneal (i.p.) injection of streptozotocin (Sigma) at 40 mg/kg of body weight per day for 5 consecutive days [22]. STZ working solution was freshly prepared by dissolving STZ in 25 mM sodium citrate solution (pH 4.0) and immediately injected after each preparation. Blood glucose levels were monitored until 4 weeks after the last dose of STZ injections.

### 2.4. Physiological Measurements

Blood glucose levels were assayed from tail vein blood by a glucose meter (OneTouch, Lifescan). Blood serum insulin levels were measured by ELISA using a mouse insulin kit (Crystal Chem). Serum glucagon levels were determined by a RIA Kit (Linco).

### 2.5. Immunostaining

BrdU at 100 mg/kg of body weight was i.p. injected into mice 16 to 24 hours before they were sacrificed. Two to three paraffin-embedded pancreas sections (5 *μ*m, at least 100 *μ*m apart) of each mouse and genotype were stained with affinity-purified antibodies against menin (1 : 500) [25], insulin (Abcam, 1 : 100), glucagon (Abcam, 1 : 100), BrdU (Accurate Chemical & Scientific, 1 : 150), Pdx1 (Millipore, 1 : 500), and GLUT2 (Millipore, 1 : 200). Secondary antibodies used were FITC (Abcam, 1 : 200) and Alexa Fluor 546 (Invitrogen, 1 : 200). Images were captured by a Nikon Eclipse E800 fluorescence microscope with a CCD digital camera. Total insulin staining area was quantified by MetaMorph software (Molecular Devices).

### 2.6. Statistical Analysis

Results are expressed as mean ± SEM. For a two-group comparison, unpaired Student's *t*-test or rank sum test was used. *P* values less than .05 were considered statistically significant.

## 3. Results

### 3.1. *Men1 *Ablation Renders Mice Resistant to STZ-Induced Hyperglycemia

**Figure 1:**
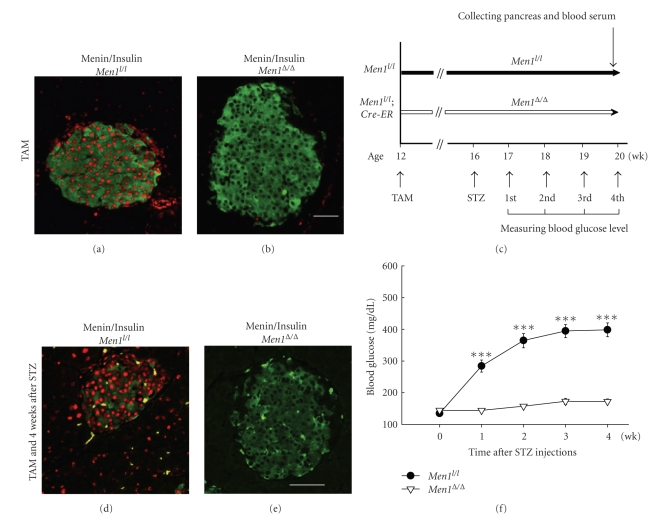
Ablation of floxed* Men1* prevents development of streptozotocin- (STZ-) induced hyperglycemia. (a) and (b) Immunostaining for menin (red) and insulin (green), respectively, in pancreatic islets in mice fed TAM and without STZ treatment. Control *Men*1^*l*/*l*^ and *Men*1^*l*/*l*^; *Cre-ER *mice (*n* = 8 mice) were fed tamoxifen (TAM) at the age of 12 weeks at 200 mg/kg of body weight per day for two consecutive days, followed by one day off and then for another two consecutive days, as described in Section 2. Four weeks after the last dose of TAM feeding, mice were sacrificed and pancreas sections of *Men*1^*l*/*l*^ (a) or *Men*1^*l*/*l*^; *Cre-ER *mice (b) were immunostaed for menin and insulin. (c) A schematic of experimental design. Control *Men*1^*l*/*l*^ (*n* = 18 mice) and *Men*1^*l*/*l*^; *Cre-ER *mice (*n* = 21 mice) were fed TAM at the age of 12 weeks. Four weeks after the last dose of TAM feeding, STZ was i.p. injected at 40 mg/kg of body weight per day for 5 consecutive days. Blood glucose levels were monitored till 4 weeks after STZ injections. (d) and (e) Immunostaining for menin (red) and insulin (green) in islets in control *Men*1^*l*/*l*^ (d) and *Men*1^*l*/*l*^; *Cre-ER* mice (e) that were treated with TAM and followed by STZ injections, as described in Figure 1(c). The pancreata were collected 4 weeks after STZ treatment. (f) Non-fasting blood glucose levels before and until 4 weeks after STZ injections (*n* = 18 to 21 mice). Scale bar, 25 *μ*m. ***, *P* < .001.

To determine the impact of *Men1* excision on the development of diabetes, we evaluated whether *Men1* excision can prevent streptozotocin- (STZ-) induced hyperglycemia using a conditional *Men1* knockout mouse model. To generate *Men*1^*l*/*l*^; *Cre-ER* mice, mice harboring floxed *Men1* were crossed with mice harboring an Ubc9 promoter-driven *Cre-ERT2 *transgene (*Cre-ER*) [17, 24]. The resulting *Men*1^*l*/+^; *Cre-ER *mice and *Men*1^*l*/+^ mice were bred, and *Men*1^*l*/*l*^; *Cre-ER* mice and their littermates (*Men*1^*l*/*l*^) were used for the experiments. To check the efficiency of *Men1 *excision in *Men*1^*l*/*l*^; *Cre-ER *mice, *Men*1^*l*/*l*^ and *Men*1^*l*/*l*^; *Cre-ER* mice were fed TAM, and menin levels in pancreatic islets were determined by immunofluorescence staining 4 weeks after TAM feeding. Immunostaining showed that in the control *Men*1^*l*/*l*^ mice, menin was preferentially expressed in the nucleus of islet cells as compared with acinar cells (Figure 1(a)). Menin was barely detectable in both islets and acinar cells of the *Men*1^*l*/*l*^; *Cre-ER* mice, indicating that the *Men1 *gene was effectively excised in *Men*1^*l*/*l*^; *Cre-ER* mice (Figure 1(b)). 

We then administered multiple low-dose STZ (MLD-STZ) to the control and *Men1*-excised mice to induce beta cell damage and diabetes four weeks after *Men1* excision (Figure 1(c)) [19, 22]. Immunofluorescence staining showed that *Men1 *was effectively excised in the islets of *Men*1^*l*/*l*^; *Cre-ER* mice as compared with control mice 4 weeks after STZ treatment (Figures 1(d) and 1(e)). In the control mice, nonfasting blood glucose levels started to increase as early as one week after STZ injections and ultimately reached 400 mg/dL 4 weeks after STZ injections (Figure 1(f)). *Men1*-excised mice, on the other hand, did not develop hyperglycemia during the whole period of study (Figure 1(f)), demonstrating that *Men1* ablation prevents STZ-induced hyperglycemia.

### 3.2. *Men1 *Ablation Promotes Beta-Cell Proliferation and Increases the Number of Functional Beta Cells

To determine the underlying mechanisms whereby *Men1* ablation prevents STZ-induced hyperglycemia, we determined cell composition in islets, BrdU incorporation by beta cells, islet size, and circulating insulin levels in control and *Men1*-excised mice 4 weeks after STZ injections.

**Figure 2:**
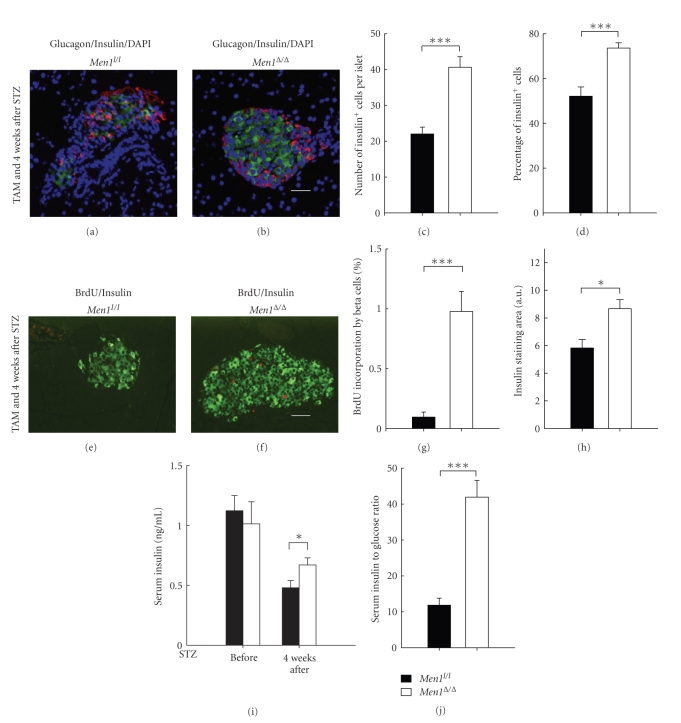
Ablation of floxed* Men1* promotes beta-cell proliferation and increases beta cell number in STZ-treated mice. Study design was described in Figure 1(c). Control *Men*1^*l*/*l*^ (*n* = 18 mice) and *Men*1^*l*/*l*^; *Cre-ER *mice (*n* = 21 mice) were fed TAM at age of 12 weeks. Four weeks after the last dose of TAM feeding, STZ was i.p. injected at 40 mg/kg of body weight per day for 5 consecutive days. Pancreata and blood serum were collected from mice for further analysis, 4 weeks after STZ injections. (a-b) Immunostaining for insulin (green) and glucagon (red) in islets in control *Men*1^*l*/*l*^ (a) and *Men*1^*l*/*l*^; *Cre-ER* mice (b). Nuclei were counterstained using DABI (blue). (c) Quantitation of number of insulin-secreting beta cells in islets (*n* = 8 mice). (d) Quantitation of percentage of beta cells in islets (*n* = 8 mice). **(**e) and (f) Immunostaining for BrdU (red) and insulin (green) in islets in control *Men*1^*l*/*l*^ (e) and *Men*1^*l*/*l*^; *Cre-ER* mice (f). (g) Quantitation of BrdU incorporation by beta cells (*n* = 8 mice). (h) Quantitation of insulin staining area (*n* = 8 mice). (i) Non-fasting serum insulin levels before and 4 weeks after STZ injections (*n* = 18 to 21 mice). (j) Ratio of serum insulin (ng/mL) to blood glucose levels (mg/dL), multiplied by 10000. Scale bar, 25 *μ*m. *, *P* < .05; ***, *P* < .001.

Immunofluorescence staining showed that both the number and the percentage of insulin-producing beta cells were significantly reduced in islets of the control mice (Figures 2(a), 2(c)-2(d)), as compared with the *Men1*-excised mice (Figures 2(b)–2(d)). These findings suggest that *Men1 *excision either protected beta cells from STZ-induced beta cell damage or increased the number of functional beta cells. BrdU incorporation by beta cells, a marker for cell proliferation, was significantly higher in the *Men1*-excised mice than the control mice (Figures 2(e)-2(g)), indicating that *Men1* excision increased the number of beta cells at least partly through promoting beta-cell proliferation. Consistent with an increase in the number of proliferating beta cells after *Men1 *excision, insulin staining area was significantly larger in the *Men1*-excised mice (Figure 2(h)). Random blood insulin levels, which were similar between the control and *Men1*-excised mice before STZ treatment, dropped in both groups after STZ injections (Figure 2(i)). However, it is noteworthy that blood insulin level was reduced by *∼*57% in the control *Men1*-expressing mice (before, 1.12 ng/mL versus 4 wk after, 0.48 ng/mL, Figure 2(i)), while it was only reduced by *∼*34% in the *Men1*-excised mice (before, 1.01 ng/mL versus 4 wk after, 0.67 ng/mL, Figure 2(i)), suggesting that there was less STZ-induced beta cell damage and deficit in insulin production and/or secretion in the *Men1*-exicsed mice. Moreover, the blood insulin level was significantly higher in the *Men1*-excised mice than the control mice after STZ injections (*P* < .05, Figure 2(i)).

 It is also noteworthy that the control mice, even with severe hyperglycemia (*∼*400 mg/dL, Figure 1(f)), did not secrete more insulin to reduce high blood glucose levels (4 wk after STZ, 0.48 ng/mL, Figure 2(i)), indicating a very obvious deficit in insulin production and/or secretion in the control mice after STZ treatment. On the contrary, even though the blood insulin level in the *Men1*-excised mice was lower four weeks after than before STZ injections (before STZ, 1.01 ng/mL; 4 wk after STZ, 0.67 ng/mL, Figure 2(i)), the *Men1*-excised mice were normoglycemic (*∼*150 mg/dL, Figure 1(f)). These results indicate that insulin produced/secreted by the* Men1*-excised mice was adequate to control their blood glucose levels within a normal range and the nearly normal blood glucose levels did not trigger further insulin secretion/production. Notably, we also found that the ratio of blood insulin to glucose levels was significantly higher in the *Men1*-excised mice than the control *Men1*-expressing mice (Figure 2(j)), suggesting that, after STZ treatment, islet function and/or total insulin secretion by islets in response to blood glucose stimulation was better preserved in the *Men1*-excised mice, and that beta cells in the *Men1*-excised mice were able to secrete more insulin into blood circulation and keep blood glucose levels within a relatively normal range. Taken together, these findings strongly suggest that *Men1* ablation prevents STZ-induced hyperglycemia at least in part through promoting beta-cell proliferation, resulting in the maintenance of a larger number of functional beta cells and higher circulating insulin concentrations.

**Figure 3:**
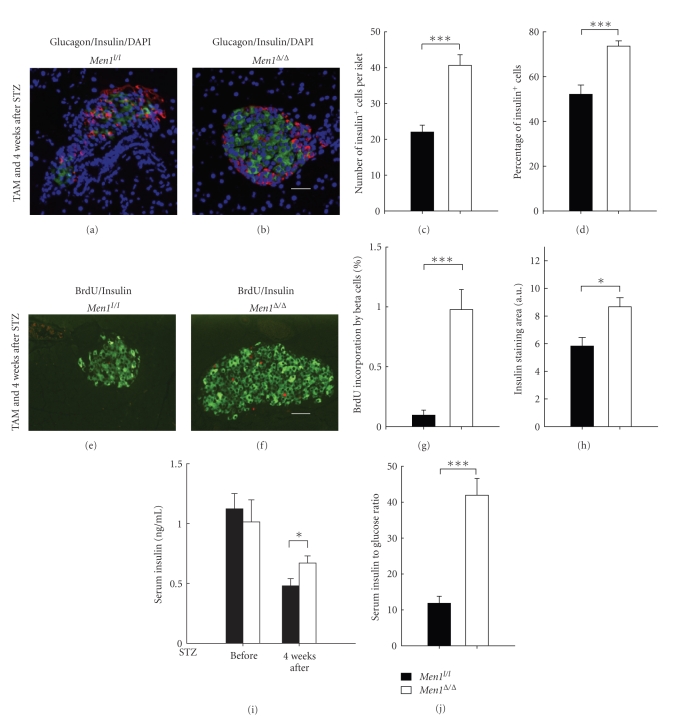
Ablation of floxed* Men1* does not affect the number of glucagon-secreting alpha cells and serum glucagon levels in STZ-treated mice. Study design was described in Figure 1(c). Control *Men*1^*l*/*l*^ (*n* = 18 mice) and *Men*1^*l*/*l*^; *Cre-ER *mice (*n* = 21 mice) were fed TAM at age of 12 weeks. Four weeks after the last dose of TAM feeding, STZ was i.p. injected at 40 mg/kg of body weight per day for 5 consecutive days. Pancreata and blood serum were collected from mice for further analysis, 4 weeks after STZ injections. (a) Quantitation of number of alpha cells in islets (*n* = 8 mice). (b) Quantitation of the percentage of alpha cells in islets (*n* = 8 mice). (c) Non-fasting serum glucagon levels before and 4 weeks after STZ injections (*n* = 18 to 21 mice). *, *P* < .05.

It has been reported that the number of glucagon-producing alpha cells is increased in diabetic state, and knockdown of glucagon-receptor normalizes high glucose levels in diabetic mice [26, 27]. Recent studies have also shown that *Men1*-excised glucagon-producing alpha cells can transdifferentiate to insulin-producing beta cells in normal mice, and alpha cells transdifferentiate into beta cells more rapidly when beta cells are under severe stress [28, 29]. Therefore, to determine whether *Men1* ablation prevents STZ-induced hyperglycemia through reducing the number of alpha cells, the average number and percentage of alpha cells in islets and circulating glucagon levels were determined. We found that the number of glucagon-secreting alpha cells per islet was similar in the control and *Men1*-excised mice (Figure 3(a)). Although the percentage of alpha cells was higher in control islets than *Men1*-excised islets after STZ treatment (Figure 3(b)), which was likely due to a low number of beta cells and consequently a lower total number of cells in islets in the control mice, the circulating glucagon levels were similar between the control and *Men1*-excised mice (Figure 3(c)). This result suggests that transdifferentiation of alpha cells into beta cells was not substantially affected by *Men1* excision 4 weeks after STZ injections. Therefore, *Men1* ablation likely prevents STZ-induced hyperglycemia mainly through its impact on beta-cell proliferation and increasing the number of functional beta cells.

### 3.3. Men1 Ablation Specifically in Pancreatic Beta Cells Prevents Development of STZ-Induced Hyperglycemia

**Figure 4:**
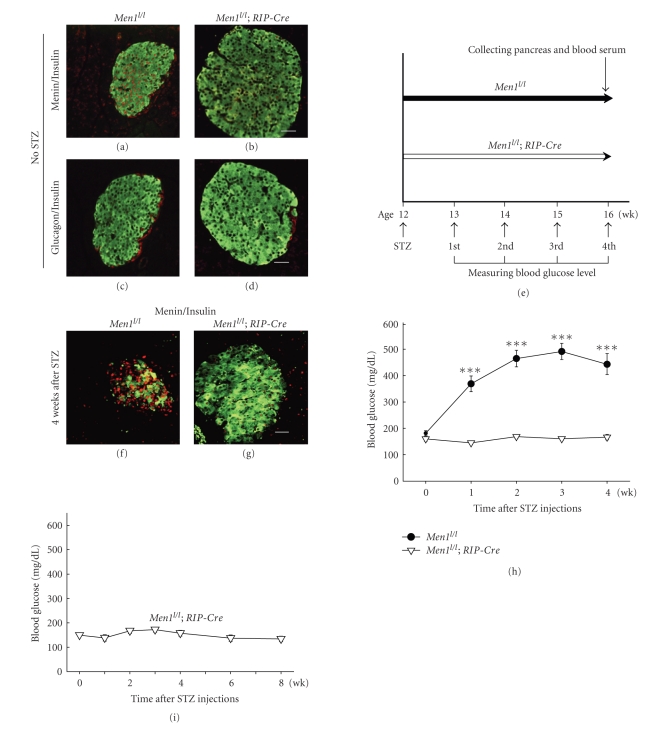
Tissue specific ablation of floxed* Men1* in beta cells increases resistance to STZ-induced hyperglycemia. (a–d) Control *Men*1^*l*/*l*^ and *Men*1^*l*/*l*^; *RIP-Cre* mice at the age of 12 weeks (*n* = 2 to 3 mice) without STZ treatment were sacrificed, and pancreata sections were immunostainined for menin, insulin, or glucagon. (a-b) Immunostaining for menin (red) and insulin (green) in islets in control *Men*1^*l*/*l*^ (a) and *Men*1^*l*/*l*^; *RIP-Cre* mice (b). (c-d) Immunostaining for insulin (green) and glucagon (red) in islets in *Men*1^*l*/*l*^ (c) and *Men*1^*l*/*l*^; *RIP-Cre* mice (d). (e) A schematic of experimental design. Control *Men*1^*l*/*l*^ and *Men*1^*l*/*l*^; *RIP-Cre* mice (*n* = 15 mice) at the age of 12 weeks were injected with STZ at 40 mg/kg of body weight per day for 5 consecutive days. Blood glucose levels were monitored, and mice were sacrificed 4 weeks after STZ injections. (f-g) Immunostaining for menin (red) and insulin (green) in islets in *Men*1^*l*/*l*^ (f) and *Men*1^*l*/*l*^; *RIP-Cre* mice (g) after STZ injections, as described in Figure 4(e). The pancreata were collected 4 weeks after STZ treatment. (h) Non-fasting blood glucose levels before and 4 weeks after STZ injections (*n* = 12 to 15 mice). (i) *Men*1^*l*/*l*^; *RIP-Cre* mice at the age of 12 weeks were injected with STZ at 40 mg/kg of body weight per day for 5 consecutive days. Blood glucose levels in *Men*1^*l*/*l*^; *RIP-Cre* mice before and until 8 weeks after STZ injections (*n* = 6 mice).

To determine whether *Men1* excision, specifically in beta cells, contributes to prevention of STZ-induced hyperglycemia, *Men*1^*l*/*l*^; *RIP-Cre* mice were generated where *Men1* was preferentially excised in beta cells by the rat insulin promoter-driven *Cre* recombinase [15]. Immunostaining showed that menin protein level was significantly reduced exclusively in beta cells but not in adjacent acinar cells in the *Men*1^*l*/*l*^; *RIP-Cre* mice at 12 weeks of age (Figure 4(b)), as compared with the control *Men*1^*l*/*l*^ mice (Figure 4(a)). These findings suggest that *Men1* was excised specifically in beta cells in the *Men*1^*l*/*l*^; *RIP-Cre* mice. Islet size was increased in the *Men*1^*l*/*l*^; *RIP-Cre *mice as compared with the control *Men*1^*l*/*l*^ mice (Figures 4(a) and 4(b)), which was consistent with the observations in the previous study [15]. On the other hand, composition of beta cells and alpha cells in islets was similar between the control and *Men*1^*l*/*l*^; *RIP-Cre *mice (Figures 4(c) and 4(d)). 

**Figure 5:**
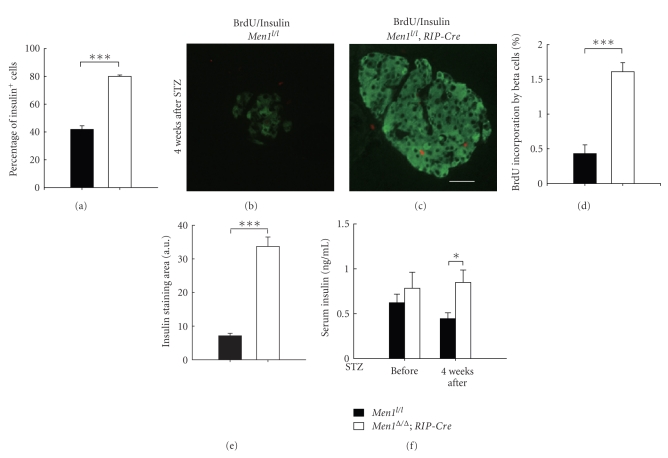
Tissue specific ablation of floxed* Men1* promotes beta-cell proliferation and increases beta cell number in STZ-treated mice. Study design was described in Figure 4(e). Control *Men*1^*l*/*l*^ and *Men*1^*l*/*l*^; *RIP-Cre* mice (*n* = 15 mice) at the age of 12 weeks were injected with STZ at 40 mg/kg of body weight per day for 5 consecutive days. Pancreata and blood serum were collected from mice for further analysis, 4 weeks after STZ injections. (a) Quantitation of percentage of insulin-secreting beta cells in islets (*n* = 4 to 7 mice). (b and c) Immunostaining for BrdU (red) and insulin (green) in islets in *Men*1^*l*/*l*^ (b) and *Men*1^*l*/*l*^; *RIP-Cre* mice (c). (d) Quantitation of BrdU incorporation by beta cells (*n* = 4 to 7 mice). (e) Quantitation of insulin staining area (*n* = 4 to 7 mice). (f) Non-fasting serum insulin levels before and 4 weeks after STZ injections (*n* = 12 to 15 mice). Scale bar, 25 *μ*m. *, *P* < .05; ***, *P* < .001.

*Me* *n*1^*l*/*l*^ and *Men*1^*l*/*l*^; *RIP-Cre* mice at 12 weeks of age were then injected with MLD-STZ, and blood glucose levels were monitored up to 4 weeks after STZ injections (Figure 4(e)). *Men1 *was effectively excised in islets of the *Men*1^*l*/*l*^; *RIP-Cre *mice as compared with the control mice (Figures 4(f) and 4(g)). Similar to what was observed in the TAM-treated *Men*1^*l*/*l*^; *Cre-ER* mice, specific *Men1* excision in beta cells in the *Men*1^*l*/*l*^; *RIP-Cre* mice also rendered mice resistant to STZ-induced beta cell damage and hyperglycemia. Non-fasting blood glucose levels in the control mice started to increase as early as one week after STZ injections and ultimately reached 450 mg/dL 4 weeks after STZ injections (Figure 4(h)). In contrast, *Men*1^*l*/*l*^; *RIP-Cre* mice did not develop hyperglycemia during the whole study (Figure 4(h)). Moreover, blood glucose levels in the *Men*1^*l*/*l*^; *RIP-Cre* mice remained normal up to 8 weeks after STZ injections (Figure 4(i)). Consistent with their normal blood glucose levels 4 weeks after STZ injections, the percentage of insulin-secreting beta cells in islets was significantly higher in the *Men*1^*l*/*l*^; *RIP-Cre *mice than the control mice (Figure 5(a)), suggesting that there was less beta cell damage and/or more functional beta cells preserved in the *Men*1^*l*/*l*^; *RIP-Cre *mice. BrdU incorporation by beta cells was also significantly higher in the *Men1*; *RIP-Cre* mice than the control mice (Figures 5(b)–5(d)), further confirming that *Men1* excision increased the number of beta cells at least partly through promoting beta-cell proliferation. Consistent with the observation that beta-cell proliferation was increased after *Men1 *excision (Figures 5(b)–5(d)), the insulin staining area was significantly larger in the *Men*1^*l*/*l*^; *RIP-Cre *mice than the control mice (Figure 5(e)). Furthermore, random serum insulin levels in the *Men*1^*l*/*l*^; *RIP-Cre *mice was significantly higher than the control mice after STZ injections (Figure 5(f)). Moreover, there was no much difference in blood insulin levels in the *Men*1^*l*/*l*^; *RIP-Cre* mice before and 4 weeks after STZ injections (Figure 5(f)), strongly suggesting that there was less beta cell damage and consequently higher circulating insulin levels in the *Men*1^*l*/*l*^; *RIP-Cre* mice. These results also support that *Men1*-specific excision in pancreatic beta cells prevents STZ-induced hyperglycemia.

### 3.4. Men1 Ablation Preserves GLUT2 Membrane Localization in Pancreatic Beta Cells in STZ-Treated Mice

Pdx-1 is a crucial transcription factor for the development and regeneration of beta cells in both normal and diabetic mice [30, 31]. GLUT2 is an important beta cell membrane protein responsible for transporting glucose across beta cell membranes, and it is involved in glucose sensing and glucose-stimulated insulin secretion [32]. STZ binds to GLUT2 in beta cell membranes and is transported into beta cells, and it induces beta cell damage through several mechanisms including damaging genomic DNA [20]. In order to determine whether *Men1* ablation prevents STZ-induced hyperglycemia partly through its regulation on Pdx-1 and/or GLUT2, we sought to detect expression or localization of Pdx-1 and GLUT2 in beta cells in STZ-treated control and *Men1*-excised mice by immunostaining.

**Figure 6:**
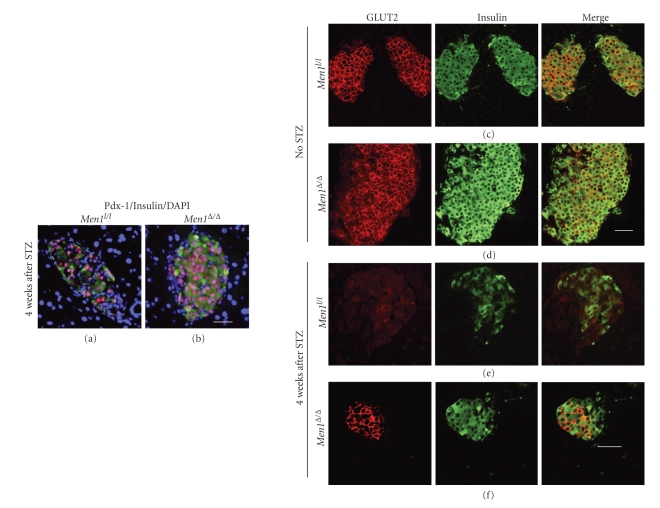
Ablation of floxed* Men1* preserves membrane localization of glucose transporter 2 (GLUT2) in beta cells in STZ-treated mice. (a-b) Immunostaining for Pdx-1 (red) and insulin (green) in islets in control *Men*1^*l*/*l*^ (a) and *Men*1^*l*/*l*^; *Cre-ER* mice (b) treated with TAM, followed by STZ injections, as described in Figure 1(c). Control *Men*1^*l*/*l*^ (*n* = 18 mice) and *Men*1^*l*/*l*^; *Cre-ER *mice (*n* = 21 mice) were fed TAM at age of 12 weeks. Four weeks after the last dose of TAM feeding, STZ was i.p. injected at 40 mg/kg of body weight per day for 5 consecutive days. Pancreata were collected from mice 4 weeks after STZ injections. Nuclei were counterstained using DABI (blue). (c-d) Immunostaining for GLUT2 (red) and insulin (green) in islets in control *Men*1^*l*/*l*^ (c) and *Men*1^*l*/*l*^; *Cre-ER *mice (d) without STZ treatment, as described in Figures 1(a) and 1(b). Control *Men*1^*l*/*l*^ and *Men*1^*l*/*l*^; *Cre-ER *mice (*n* = 8 mice) were fed tamoxifen (TAM) at the age of 12 weeks at 200 mg/kg of body weight per day for two consecutive days, followed by one day off and then for another two consecutive days. Pancreata were collected 4 weeks after TAM feeding. (e-f) Immunostaining for GLUT2 and insulin in islets in control *Men*1^*l*/*l*^ (e) and *Men*1^*l*/*l*^; *Cre-ER* mice (f) treated with TAM and followed by STZ injections, as described in Figure 1(c). Control *Men*1^*l*/*l*^ (*n* = 18 mice) and *Men*1^*l*/*l*^; *Cre-ER *mice (*n* = 21 mice) were fed TAM at age of 12 weeks. Four weeks after the last dose of TAM feeding, STZ was i.p. injected at 40 mg/kg of body weight per day for 5 consecutive days. Pancreata were collected 4 weeks after STZ injections. Scale bar, 25 *μ*m.

We found that the number of Pdx-1 positive cells was higher in *Men1*-excised islets than control islets (Figures 6(a) and 6(b)). However, given the fact that Pdx-1 protein levels in individual beta cells did not appear substantially different between control and *Men1*-excised islets after STZ injections, whether *Men1* excision directly up-regulates *Pdx-1 *expression still needs to be determined. Immunofluorescence staining showed that GLUT2 was detectable in both cell membranes and cytoplasm of beta cells in control and *Men1*-excised mice without STZ treatment (Figures 6(c) and 6(d)). Notably, after STZ injections, GLUT2 expression was largely preserved in beta cell membranes in the *Men1*-excised mice (Figure 6(f)), while it was barely detectable in beta cell membranes in the control mice (Figure 6(e)). These findings suggest that *Men1* excision protects/preserves GLUT2 membrane localization in beta cell membranes and thus preserves the function of beta cells after STZ injections.

## 4. Discussions and Conclusions

Previous studies have shown that beta-cell proliferation and circulating insulin levels were increased in mice long after *Men1* excision [15, 17]. However, whether relatively acute *Men1 *excision can prevent STZ-induced hyperglycemia and its underlying mechanisms remains unclear. In the present study, we employed an approach of temporally controlled *Men1* excision in multiple tissues as well as beta-cell specific *Men1* excision and found that *Men1* excision prevented development of hyperglycemia in STZ-treated mice. Our results further showed that *Men1* excision prevented development of hyperglycemia mainly through increasing the number of functional beta cells. 

It was also noticed that, in contrast to the observation that blood insulin level was reduced in the *Men*1^*l*/*l*^ *; Cre-ER* mice subjected to STZ treatment 4 weeks after *Men1* excision, blood insulin level was not decreased in the STZ-treated *Men*1^*l*/*l*^ *; RIP-Cre* mice (*Men*1^*l*/*l*^ *; Cre-ER* mice, Figure 2(i); *Men*1^*l*/*l*^ *; RIP-Cre* mice, Figure 5(f)). The difference in the time and duration of *Men1* excision in these two groups of mice might partially explain the discrepancy. For the *Men*1^*l*/*l*^ *; Cre-ER* mice, the *Men1* gene was excised at age of 12 weeks through TAM feeding and these mice were then challenged with MLD-STZ 4 weeks after the last dose of TAM feeding (Figure 1(c)). There were only 4 weeks (after the last dose of TAM) for *Men1*-excised mice to replicate or regenerate more functional beta cells before they were injected with STZ. In contrast, in the *Men*1^*l*/*l*^ *; RIP-Cre* mice, the *Men1* gene was excised in beta cells during embryonic development, since the rat insulin promoter-driven *Cre* recombinase is expressed and activated by insulin during embryonic stage. There were about 12 weeks for *Men*1^*l*/*l*^ *; RIP*-*Cre* mice to produce more functional beta cells before they were challenged with STZ. Duration of *Men1* excision was much longer in the *Men*1^*l*/*l*^ *; RIP-Cre* mice than the TAM-treated *Men*1^*l*/*l*^ *; Cre-ER* mice, therefore it is likely that the *Men*1^*l*/*l*^ *; RIP-Cre *mice have more functional beta cells than the TAM-treated *Men*1^*l*/*l*^ *; Cre-ER* mice before STZ injections and consequently higher number of beta cells after STZ injections. As a result, blood insulin level was not significantly reduced in the *Men*1^*l*/*l*^ *; RIP-Cre *mice after STZ injections. In addition, it is also possible that earlier *Men1* excision during embryonic stage might lead to some compensation or adaptation of beta cells in the *Men*1^*l*/*l*^ *; RIP-Cre* mice, which could also partially contribute to the relative resistance of beta cells to STZ-induced damage and reduction in blood insulin level.

Recent studies have shown that *Men1*-excised glucagon-producing alpha cells transdifferentiate to insulin-producing beta cells in mice [28]. Moreover, transdifferentiation from alpha cells to beta cells occurs more rapidly when beta cells are under severe stress [29]. However, our results showed that the average number of alpha cells per islet and blood glucagon level were similar between the control and *Men1*-excised mice, suggesting that *Men1* excision did not substantially trigger transdifferentiation of alpha cells to beta cells 4 weeks after STZ injections. However, we cannot rule out that *Men1*-excised alpha cells or other non-beta cells in the pancreas can reprogram into insulin-secreting beta-cells during the late stage of STZ-induced diabetes. 

Our further analysis using immunostaining revealed that there were more Pdx-1 positive cells in *Men1*-excised mice. Pdx-1 is an important transcription factor in beta-cell development and growth [33, 34]. Our results showed that Pdx-1 protein level in an individual beta-cell does not appear to increase significantly in *Men1*-excised mice as compared with control mice, suggesting that Pdx-1 expression level might be a marker for functional beta-cells after STZ injections, and a reduced number of Pdx-1 positive cells in the control mice may be a result of reduction in the total number of functional beta-cells in those mice. Immunostaining on pancreas sections also showed that GLUT2 localization in beta-cell membranes after STZ injections were largely preserved in the *Men1*-excised mice as compared with the control mice (Figures 6(e) and 6(f)). This result may partly explain why *Men1*-excised mice showed normal blood glucose levels and higher circulating insulin levels, since GLUT2 is involved in glucose sensing and glucose-stimulated insulin secretion [32]. On the other hand, because *Men1 *excision did not reduce membrane expression of GLUT2 in beta-cells in mice without STZ treatments (Figures 6(c) and 6(d)), it is unlikely that relative resistance of *Men1*-excised mice to STZ-induced beta-cell damage was due to changes in membrane GLUT2 expression in beta-cells. Alternatively, we could not rule out that *Men1* excision improves the overall function of beta-cells, rendering *Men1*-excised cells more resistant to STZ-induced beta-cell damage. 

It is also noteworthy that the *RIP-Cre* transgene, which we used to specifically excise *Men1* in beta-cells in *Men*1^*l*/*l*^; *RIP-Cre* mice, has been reported to express in non-beta cells, including the lung, spleen, testis, and brain [35], especially in a group of poorly defined neurons in the hypothalamus (*RipCre* neurons) [36]. Knockout of insulin receptor substrate 2 in this group of hypothalamic neuronal population causes disruption in energy homeostasis, increases food intake, and leads to obesity at a later stage [36]. Because it has not been reported that tumors develop in the hypothalamus in either MEN1 patients or *Men1* knockout mice, it is not clear whether menin represses proliferation of *RipCre* neurons. Moreover, it is also unclear whether *Men1* excision in *RipCre *neurons affects food intake or other pathways involved in energy homeostasis or glucose metabolism. Therefore, we could not rule out the possibility that *Men1* excision in non-beta cells may partly attribute to improvement in glucose homeostasis in STZ-treated mice. 

In conclusion, we have found that *Men1* excision prevents STZ-induced hyperglycemia mainly through increasing the number of functional beta cells. BrdU incorporation by beta cells, islet size, and circulating insulin levels were significantly increased in *Men1*-excised mice. Consistent with our findings, menin is physiologically suppressed through the prolactin signaling pathway in pancreatic beta cells in pregnant mice to prevent gestational diabetes [37]. These results suggest that menin might be targeted to promote beta-cell proliferation and ameliorate diabetes. However, since cell hyperplasia, insulinoma, and hyperinsulinemia have been found in *Men1* knockout mouse models or MEN1 patients [15, 38, 39], there is a justifiable concern that disruption of menin aiming to promote beta-cell proliferation may lead to insulinoma, and permanent deletion or inhibition of menin itself might not be a viable therapeutic approach to treat diabetes. Instead, it might be possible to promote beta-cell proliferation through reversibly inhibiting menin or blocking the interaction between menin and its partners. These approaches may retain menin's tumor suppressing functions, such as DNA repair [40], but transiently dampen its ability to suppress beta-cell proliferation, thereby increasing the number of functional endogenous beta cells and ameliorating diabetes. In this regard, small molecule compounds that target the interaction between menin and its partners, if developed, could be useful for preventing or treating diabetes.

##  Conflict of Interests

Relevant to this paper no potential conflict of interests is declared by the authors.
